# Risk of pregnancy complications and adverse birth outcomes after maternal A(H1N1)pdm09 influenza: a Norwegian population-based cohort study

**DOI:** 10.1186/s12879-018-3435-8

**Published:** 2018-10-22

**Authors:** Ida Laake, Gro Tunheim, Anna Hayman Robertson, Olav Hungnes, Kristian Waalen, Siri E. Håberg, Siri Mjaaland, Lill Trogstad

**Affiliations:** 1 Department of Infectious Disease Epidemiology and Modelling, Norwegian Institute of Public Health, Oslo, Norway; 2 Department of Infectious Disease Immunology, Norwegian Institute of Public Health, Oslo, Norway; 3 K.G. Jebsen Center for Influenza Vaccine Research, University of Oslo, Oslo, Norway; 4 Department of Influenza, Norwegian Institute of Public Health, Oslo, Norway; 5 Division for Mental and Physical Health, Norwegian Institute of Public Health, Oslo, Norway

**Keywords:** Influenza, Pandemic, A(H1N1)pdm09, Antibodies, Pregnancy, Birth outcomes

## Abstract

**Background:**

The effects of maternal influenza infection on the fetus remain unclear. We studied mild influenza and influenza antibodies in relation to birth weight and risks of pre-eclampsia, preterm birth (PTB), and small for gestational age (SGA) birth among the unvaccinated participants in the Norwegian Influenza Pregnancy Cohort.

**Methods:**

Pregnant women attending a routine ultrasound were recruited from four hospitals in Norway shortly after the 2009 A(H1N1) pandemic. The present study was restricted to unvaccinated participants who were pregnant during the pandemic. Information on the participants was obtained through questionnaires and linkage with national registries. Maternal blood samples were collected at delivery. Women with laboratory-confirmed A(H1N1)pdm09 influenza, a clinical diagnosis of influenza, or self-reported influenza during the pandemic were classified as having had influenza. A(H1N1)pdm09-specific antibodies in serum were detected with the hemagglutination-inhibition assay. Detection of antibodies was considered an indicator of infection during the pandemic in the unvaccinated participants. Odds ratios were estimated with logistic regression. Quantile regression was used to estimate differences in the distribution of birth weight.

**Results:**

Among the 1258 women included in this study, there were 37 cases of pre-eclampsia, 41 births were PTB, and 103 births were SGA. 226 women (18.0%) had influenza during the pandemic. The majority of cases did not receive medical care, and only a small proportion (1.3%) of the cases were hospitalized. Thus, the cases consisted primarily of women with mild illness. No significant associations between influenza and risk of pre-eclampsia, PTB, or SGA birth were observed. Detection of A(H1N1)pdm09-specific antibodies was associated with a lower 10th percentile of birth weight, β = − 159 g (95% CI − 309, − 9).

**Conclusions:**

Mild influenza illness during pregnancy was not associated with increased risk of pre-eclampsia, PTB or SGA birth. However, influenza infection during pregnancy may reduce the birth weight of the smallest children.

## Background

Influenza may cause severe illness and death in pregnant women. During the A(H1N1) influenza pandemic in 2009, pregnant women with influenza had higher risk of hospitalization than non-pregnant individuals with influenza [1, 2]. Less is known about the effects of maternal influenza infection on the fetus. Maternal infections may increase the risk of pre-eclampsia [3], a major cause of intrauterine growth restriction and preterm birth (PTB), but there are few studies on influenza and risk of pre-eclampsia [4–7]. High rates of PTB were observed among pregnant women hospitalized with influenza during the 2009 pandemic [8–10], but these studies did not include a comparison group of pregnant women without influenza. Several other studies have included both pregnant women with and without influenza such that appropriate comparisons of the risk of adverse birth outcomes like PTB, low birth weight, or fetal death could be made [7, 11–18]. However, the number of studies on each outcome is limited, and results are inconsistent. Thus, in a recent systematic review, no firm conclusions could be drawn regarding maternal influenza in relation to the main outcomes PTB, small for gestational age (SGA) birth, and fetal death [19]. Moreover, in most studies, the influenza cases consist of women who sought medical care, thus women with mild infection are unlikely to be included among the cases. Since a substantial proportion of infected individuals develop mild illness or remain asymptomatic [20], studies on the impact of mild influenza infection on pregnancy complications and outcomes are needed.

The Norwegian Influenza Pregnancy Cohort (NorFlu) Study is a population-based cohort of women who were pregnant during the pandemic in 2009. Using this cohort, we studied maternal influenza in relation to the risk of pre-eclampsia and adverse birth outcomes among the unvaccinated participants.

## Methods

### The NorFlu study

In Norway, the main wave of the pandemic occurred from October 1, 2009 to December 31, 2009 [15]. A vaccination campaign against A(H1N1)pdm09 was started in mid-October 2009 [21]. The vaccine was recommended to all pregnant women in the second or third trimester.

The participants in the NorFlu Study were recruited from four hospitals (three in the Oslo-area and one in Bergen), where they had received the ultrasound examination offered to all pregnant women around pregnancy week 18. The ultrasound examination is usually performed at the hospital where the birth is planned. Women who had their last menstrual period between June 1, 2009 and December 1, 2009 were invited by mail to participate in the study. Recruitment took place during pregnancy week 28–40, from February 2010 to September 2010. Out of 5333 pregnant women who were invited, 3201 women (60.0%) agreed to participate. The majority of the participants were pregnant in the first or second trimester during the main wave of the pandemic. Upon return of the informed consent form that was included with the invitation, the participants were sent two questionnaires, one covering influenza and influenza vaccination and one covering pregnancy and general health. Questionnaires were completed and returned prior to delivery. Blood samples were collected at delivery from a total of 2408 (75.2%) of the participants. The protocol did not specify any exclusion criteria for blood sampling, but the capacity in the delivery unit may have prevented blood sample collection from some participants. In addition, a few women may have been unwilling to provide a blood sample. In June 2010, the influenza and vaccination questionnaire was sent to approximately 12,000 participants in another large pregnancy cohort, the Norwegian Mother and Child Cohort Study (MoBa) [22]. Women from the Oslo- or Bergen area who reported being pregnant during the past 12 months were subsequently invited to participate in the NorFlu Study. Of the 1769 invited women, 1291 gave their consent to participate. Blood samples could not be collected from these women.

The participants were linked to their records in national registries and databases by use of the unique identification number assigned to all residents of Norway. Information about the pregnancy, delivery, and infant was obtained from the Medical Birth Registry of Norway (MBRN). Records of vaccination against A(H1N1)pdm09 influenza were obtained from the Norwegian Immunization Registry. Contacts with primary care physicians that resulted in a diagnosis of influenza (R-80, International Classification of Primary Care, Second edition) were obtained from the Directorate of Health’s reimbursement database. Cases of laboratory-confirmed A(H1N1)pdm09 influenza were obtained from the Norwegian Surveillance system for Communicable Diseases.

### Outcomes

We studied the following outcomes: pre-eclampsia, PTB, SGA birth, and birth weight. Women were classified as having pre-eclampsia if mild pre-eclampsia, severe pre-eclampsia, or pre-eclampsia before week 34 had been reported to MBRN. PTB was defined as birth before 37 completed weeks of gestation. SGA birth was defined as birth weight < 10th percentile for gestational age and sex [23]. Few singletons born in Norway have birth weight < 2500 g, the World Health Organization’s definition of low birth weight. Therefore, birth weight was treated as a continuous variable.

### Definition of exposures

#### Influenza

Women were classified as having had influenza during the pandemic if they were diagnosed with influenza by a primary care physician during the main wave of the pandemic (October 1, 2009 to December 31, 2009), had laboratory confirmed A(H1N1)pdm09 influenza, or reported having influenza-like illness in October, November, or December of 2009 on the questionnaire.

#### Antibodies against a(H1N1)pdm09

Sera were analyzed for antibodies against A(H1N1)pdm09 using the hemagglutination-inhibition (HI) assay in serial two-fold dilutions, starting at dilution 1:10 [24]. HI-titer was defined as the reciprocal of the highest dilution that produced complete inhibition in the assay. The women were classified as having HI-titer ≥10 or HI-titer = 5, corresponding to an undetectable level of antibodies. The A(H1N1)pdm09 virus was antigenically very distinct from previous seasonal H1N1 viruses, and before the 2009 pandemic, the prevalence of antibodies against A(H1N1)pdm09 was very low among Norwegian women of reproductive age [25]. Since the sera in our study were collected after the pandemic, we assume that the presence of antibodies against A(H1N1)pdm09 is due to vaccination or influenza infection during the pandemic. Consequently, in unvaccinated women, detected antibodies indicate influenza infection during the pandemic.

#### Other covariates

Women who self-reported having one or more of the medical conditions that are considered risk factors for developing influenza complications (asthma, diabetes type 1, diabetes type 2, other lung diseases, obesity, cardiovascular disease, kidney disease, or impaired immune system) were considered part of an influenza risk group. Information on smoking during pregnancy was incomplete in both the general health questionnaire and the MBRN. Therefore, women were classified as smokers if they had smoked during pregnancy according to either of these sources. Start of pregnancy was set to 282 days prior to the ultrasound predicted date of birth, or the date of the last menstrual period if the ultrasound predicted date was missing.

### Study sample

**Fig. 1:**
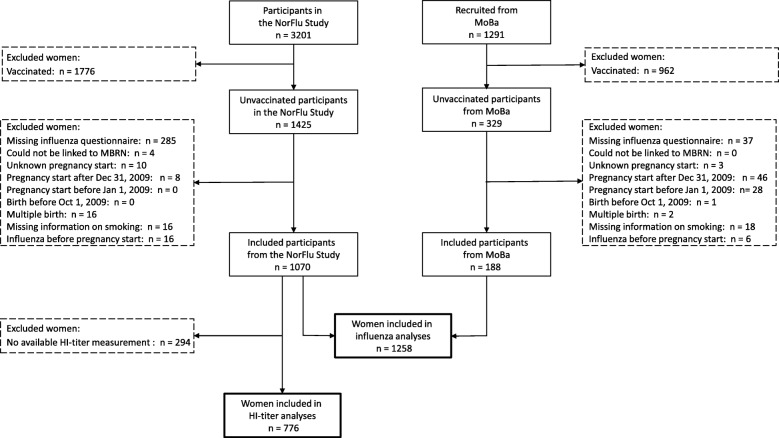
Flow chart of the participants

In total, 4492 pregnant women participated (Fig. 1). The current study was restricted to women not vaccinated against A(H1N1)pdm09. We used the influenza questionnaire in addition to the immunization registry to determine vaccination status, since approximately 10% of those vaccinated against A(H1N1)pdm09 during the pandemic were not registered in the immunization registry [26]. In total, 2738 vaccinated women were excluded (Fig. 1). Furthermore, we excluded women who did not fill out the influenza questionnaire. Women who could not be linked to the MBRN were also excluded since the outcomes were not known for these women. Women with unknown pregnancy start and women who were not pregnant during the main wave of the pandemic were also excluded. Finally, we excluded women with multiple births, missing information on smoking and women who had influenza before pregnancy. In total, 1258 women were included in the analyses with influenza as the main exposure.

Blood samples were collected from 788 of the 1258 women. Of these, 12 women were missing HI-titers. The analyses with HI-titer as the main exposure were therefore limited to the remaining 776 women with available HI-titer measurements.

### Statistical analysis

To examine influenza and HI-titer in relation to risk of pre-eclampsia, PTB, and SGA birth we used logistic regression to estimate odds ratios (ORs) with corresponding 95% confidence intervals (CIs). We used quantile regression [27] to estimate the effects of influenza and HI-titer on birth weight. For binary exposure variables, the quantile regression coefficient, β, is the difference in a specific percentile of birth weight when comparing the exposed to the unexposed. Since we were mainly interested in the smallest children, we assessed the 10th percentile and the 50th percentile (median). The analyses were adjusted for maternal age at birth (continuous), parity (primiparous/multiparous), smoking during pregnancy (yes/no), and influenza risk group (yes/no). Since the blood samples were collected at delivery, several months after the pandemic, we also adjusted the analyses with HI-titer as the main exposure for time in days between birth and the pandemic (continuous), in order to account for possible waning of the HI-titers. In the subset of women with HI-titer, no women in the influenza risk group developed pre-eclampsia. Influenza risk group was therefore removed from the model when pre-eclampsia was the outcome and HI-titer the exposure. Since we could not determine exact time of exposure to A(H1N1)pdm09 for women with HI-titer ≥10, we performed additional analyses limited to women with pregnancy start before October 1, 2009, i.e. before the main wave of the pandemic, thus excluding women who could potentially have been exposed before conception.

## Results

All births (*n* = 1258) took place in the period October 22, 2009 to October 13, 2010, but the majority (83.1%) occurred between May 2010 and August 2010. 37 women developed pre-eclampsia. Furthermore, 41 births were PTB, and 103 births were SGA. Mean gestational age was 281 days, and mean birth weight was 3587 g. 226 women (18.0%) had influenza during the pandemic. Nearly half of the influenza cases (45.6%) were medically attended, i.e. either diagnosed by a primary care physician or laboratory confirmed A(H1N1)pdm09 influenza (Table 1). The remaining 123 cases (54.4%) were based solely on self-report. Antiviral medication was used by 35 (15.5%) of the women with influenza. Only 3 women (1.3% of cases) reported that they were hospitalized with influenza. Compared to women without influenza, women with influenza were more likely to belong to an influenza risk group, less likely to be primiparous, and less likely to have smoked during pregnancy (Table 2). In addition, pregnancy start was earlier among women with influenza.

**Table 1:** Description of influenza cases, *n* = 226

Medically attended, n (%)	103 (45.6)
Influenza diagnosis (R80) from primary health care, n (%)^a^	91 (40.3)
Laboratory confirmed, n (%)^a^	38 (16.8)
Based on self-report, not medically attended, n (%)	123 (54.4)
Antiviral medication, n (%)	35 (15.5)
Hospitalized, n (%)	3 (1.3)

^a^26 cases were both diagnosed in primary health care and laboratory confirmed

**Table 2:** Characteristics of study sample by influenza status

	Without influenza, *n* = 1032	With influenza, *n* = 226
Influenza risk group (%)	5.6	13.3
Age of mother, years (mean)	32.0	31.6
No previous births, (%)	46.7	37.6
Use of folic acid during pregnancy (%)	69.6	71.2
Smoking during pregnancy (%)	4.5	1.3
Date of pregnancy start (median)	Oct 02, 2009	Sept 12, 2009
Second or third trimester at start of pandemic (%)^a^	5.6	8.4
Days between end of pandemic and birth (mean)^b^	186	170
Date of birth (median)	July 11, 2010	June 21, 2010
Recruited from the Norwegian Mother and Child Cohort Study (%)	15.1	14.2
Blood sample collected at birth (%)	61.3	68.6

^a^Start of pandemic defined as October 1, 2009

^b^End of pandemic defined as December 31, 2009

### Influenza

Using a wide definition of influenza (see above) that included both self-reported and medically attended cases, influenza during pregnancy was not significantly associated with risk of pre-eclampsia, PTB, or SGA birth among the women in our study: ORs were 1.44 (95% CI 0.64, 3.26), 0.77 (95% CI 0.32, 1.88), and 1.35 (95% CI 0.80, 2.28), respectively (Table 3). Furthermore, birth weight was similar for women with and without influenza, the difference in the 10th percentile was 22 g (95% CI − 130, 174), and the difference in the median was − 4 g (95% CI − 74, 67).

**Table 3:** Influenza in relation to risk of pre-eclampsia, preterm birth, small for gestational age birth, and birthweight

	Without influenza, *n* = 1032	With influenza, *n* = 226
Pre-eclampsia		
Cases, n (%)	29 (2.8)	8 (3.5)
Crude OR (95% CI)	1 (Ref)	1.27 (0.57, 2.81)
Adjusted OR (95% CI)^a^	1 (Ref)	1.44 (0.64, 3.26)
Preterm birth		
Cases, n (%)	35 (3.4)	6 (2.7)
Crude OR (95% CI)	1 (Ref)	0.78 (0.32, 1.87)
Adjusted OR (95% CI)^a^	1 (Ref)	0.77 (0.32, 1.88)
Small for gestational age birth		
Cases, n (%)	82 (7.9)	21 (9.3)
Crude OR (95% CI)	1 (Ref)	1.19 (0.72, 1.96)
Adjusted OR (95% CI)^a^	1 (Ref)	1.35 (0.80, 2.28)
Birth weight		
10th percentile (g)	2975	2898
Difference in 10th percentile, β (95% CI)^b^	1 (Ref)	22 (− 130, 174)
50th percentile (g)	3589	3614
Difference in 50th percentile, β (95% CI)^b^	1 (Ref)	−4 (− 74, 67)

^a^Logistic regression adjusted for maternal age at birth (continuous), parity (primiparous/multiparous), smoking during pregnancy (yes/no), and influenza risk group (yes/no)

^b^Quantile regression adjusted for maternal age at birth (continuous), parity (primiparous/multiparous), smoking during pregnancy (yes/no), and influenza risk group (yes/no)

### Antibodies against a(H1N1)pdm09

In the subsample of 776 women with measured HI-titers, the prevalence of influenza (self-reported and medically attended cases combined) during the pandemic was almost three times higher among women with HI-titer ≥10 than among women with HI-titer = 5, i.e. those with an undetectable level of antibodies (Table 4). The two groups were similar in terms of age, parity, and time of pregnancy start.

**Table 4:** Characteristics of study sample by maternal HI-titer at delivery

	HI-titer = 5, *n* = 575	HI-titer ≥10, *n* = 201
Influenza (%)	13.2	37.8
Influenza risk group (%)	7.8	8.0
Age of mother, years (mean)	32.0	31.7
No previous births (%)	53.6	48.8
Smoking during pregnancy (%)	3.3	5.0
Use of folic acid during pregnancy (%)	69.9	69.2
Date of pregnancy start (median)	Sept 29, 2009	Sept 22, 2009
Second or third trimester at start of pandemic (%)^a^	2.8	2.0
Days between end of pandemic and birth (mean)^b^	188	184
Date of birth (median)	July 08, 2010	July 03, 2010

^a^Start of pandemic defined as October 1, 2009

^b^End of pandemic defined as December 31, 2009

We observed no significant associations between HI-titer and risk of pre-eclampsia, PTB, or SGA birth: ORs were 0.83 (95% CI 0.29, 2.36), 1.26 (95% CI 0.47, 3.40), and 1.30 (95% CI 0.74, 2.81), respectively (Table 5). In contrast, we observed a close to significant difference (*p* = 0.055) in the 10th percentile of birth weight when comparing women with HI-titer ≥10 to women with HI-titer = 5, β = − 123 g (95% CI − 248, 2). Median birth weight was similar in the two groups, β = − 10 g (95% CI − 97, 76).

**Table 5:** HI-titer in relation to risk of pre-eclampsia, preterm birth, small for gestational age birth, and birth weight

	HI-titer = 5, *n* = 575	HI-titer ≥10, *n* = 201
Pre-eclampsia		
Cases, n (%)	16 (2.8)	5 (2.5)
Crude OR (95% CI)	1 (Ref)	0.89 (0.32, 2.46)
Adjusted OR (95% CI)^a^	1 (Ref)	0.83 (0.29, 2.36)
Preterm birth		
Cases, n (%)	13 (2.3)	6 (3.0)
Crude OR (95% CI)	1 (Ref)	1.33 (0.50, 3.55)
Adjusted OR (95% CI)^a^	1 (Ref)	1.26 (0.47, 3.40)
Small for gestational age birth		
Cases, n (%)	45 (7.8)	20 (10.0)
Crude OR (95% CI)	1 (Ref)	1.30 (0.75, 2.26)
Adjusted OR (95% CI)^a^	1 (Ref)	1.30 (0.74, 2.81)
Birth weight		
10th percentile (g)	3000	2932
Difference in 10th percentile, β (95% CI)^b^	1 (Ref)	−123 (−248, 2)
50th percentile (g)	3560	3580
Difference in 50th percentile, β (95% CI)^b^	1 (Ref)	−10 (− 97, 76)

^a^Logistic regression adjusted for maternal age at birth (continuous), parity (primiparous/multiparous), smoking during pregnancy (yes/no), and time in days between birth and pandemic (continuous). The model was also adjusted for influenza risk group (yes/no) except with pre-eclampsia as the outcome

^b^Quantile regression adjusted for maternal age at birth (continuous), parity (primiparous/multiparous), smoking during pregnancy (yes/no), influenza risk group (yes/no), and time in days between birth and pandemic (continuous)

In the subgroup of women with babies in the lowest decile of birthweight, gestational age was similar among those with HI-titer = 5 and those with HI-titer ≥10 (269 days vs 270 days). The prevalence of influenza in this subgroup was 20.0% for women with HI-titer = 5 and 34.6% for women with HI-titer ≥10. Compared to women with HI-titer = 5, nearly twice as many women with HI-titer ≥10 belonged to an influenza risk group (10.0% vs 19.2%).

Among women with pregnancy start before October 1, 2009 (*n* = 411), HI-titer was not significantly associated with risk of pre-eclampsia, OR = 0.98 (95% CI 0.18, 5.46), or with risk of PTB, OR = 1.30 (95% CI 0.31, 5.41) (Table 6). Women with HI-titer ≥10 had higher risk of SGA birth than women with HI-titer = 5, but this was not significant, OR = 1.75 (95% CI 0.77, 3.97). However, the 10th percentile of birth weight was significantly lower for women with HI-titer ≥10 than women with HI-titer = 5, β = − 159 g (95% CI − 309, − 9). Median birth weight was slightly lower for women with HI-titer ≥10, β = − 31 g (95% CI − 158, 96).

**Table 6:** HI-titer in relation to risk of pre-eclampsia, preterm birth, small for gestational age birth, and birth weight in women with pregnancy start before October 1, 2009

	HI-titer = 5, *n* = 293	HI-titer ≥10, *n* = 118
Pre-eclampsia		
Cases, n (%)	6 (2.0)	2 (1.7)
Crude OR (95% CI)	1 (Ref)	0.82 (0.16, 4.15)
Adjusted OR (95% CI)^a^	1 (Ref)	0.98 (0.18, 5.46)
Preterm birth		
Cases, n (%)	7 (2.4)	3 (2.5)
Crude OR (95% CI)	1 (Ref)	1.07 (0.27, 4.19)
Adjusted OR (95% CI)^a^	1 (Ref)	1.30 (0.31, 5.41)
Small for gestational age birth		
Cases, n (%)	17 (5.8)	11 (9.3)
Crude OR (95% CI)	1 (Ref)	1.67 (0.76, 3.68)
Adjusted OR (95% CI)^a^	1 (Ref)	1.75 (0.77, 3.97)
Birth weight		
10th percentile (g)	3070	2940
Difference in 10th percentile, β (95% CI)^b^	1 (Ref)	−159 (−309, −9)
50th percentile (g)	3560	3610
Difference in 50th percentile, β (95% CI)^b^	1 (Ref)	−31 (− 158, 96)

^a^Logistic regression adjusted for maternal age at birth (continuous), parity (primiparous/multiparous), smoking during pregnancy (yes/no), and time in days between birth and pandemic (continuous). The model was also adjusted for influenza risk group (yes/no) except with pre-eclampsia as the outcome

^b^Quantile regression adjusted for maternal age at birth (continuous), parity (primiparous/multiparous), smoking during pregnancy (yes/no), influenza risk group (yes/no), and time in days between birth and pandemic (continuous)

## Discussion

In this study, we investigated potentially adverse effects of maternal influenza on birth outcomes in a cohort of women that were pregnant during the influenza pandemic in 2009. The influenza cases consisted primarily of women with mild illness. The majority of cases were not medically attended, and only a small proportion of the cases were hospitalized. Neither influenza in pregnancy nor detection of maternal antibodies against A(H1N1)pdm09 at the time of delivery were significantly associated with risk of pre-eclampsia, PTB, or SGA birth. However, detection of antibodies was associated with more than 100 g lower 10th percentiles of birth weight, both overall and among women with pregnancy start before the main wave of the pandemic.

The NorFlu Study is a population-based cohort, providing a unique opportunity to study the impact of mild influenza in pregnancy. Extensive information was gathered on the participants. Through linkage with the MBRN, a national registry with data of high quality, we had complete and accurate information on the outcomes we studied. In order to capture both the milder cases of influenza and those that were medically attended, we combined information from different sources. Moreover, blood samples from a large number of women were tested for antibodies against A(H1N1)pdm09, an objective indicator of infection in unvaccinated individuals that is not influenced by recall. Serology may detect both symptomatic and asymptomatic infections, and serological surveys have been used to estimate the cumulative incidence of A(H1N1)pdm09 infection [28].

The main limitation of our study is the possible misclassification of the exposures. Due to limited capacity during the pandemic, laboratory testing of suspected A(H1N1)pdm09 influenza cases was restricted [21]. Less than 1/5 of the cases in our study, and only about 1/3 of the medically attended cases, were laboratory confirmed. Approximately half of the influenza cases were based on self-reported illness. However, the participants in our study were pregnant at the time of the pandemic, so we would expect them to recall illness more accurately than the general population. Moreover, since the questionnaire was completed prior to birth, misclassification does not depend on the outcomes we studied. Furthermore, we required that all the influenza cases, both the self-reported and the medically attended, were ill during the main wave of the pandemic, thereby reducing the likelihood that their illness was caused by infectious agents other than A(H1N1)pdm09, since this was by far the dominating respiratory virus accounting for influenza-like illness during the main wave of the pandemic in Norway [29].

Given the unpredictable nature of both influenza pandemic occurrence and pregnancy, we did not have the opportunity to collect any pre-pandemic samples. Thus, we could not identify the infected individuals according to an increase in HI-titer. Influenza seropositivity is commonly defined as HI-titer ≥40 [28], which is associated with a 50% reduced risk of influenza infection [30]. However, a substantial proportion of individuals infected during the pandemic did not become seropositive [31, 32], and the use of HI-titer of 40 as a threshold has been found to lead to an underestimate of the cumulative incidence of influenza infection during the pandemic [31]. Moreover, the prevalence of antibodies against A(H1N1)pdm09 among Norwegian women aged 20 to 40 years was very low before the 2009 pandemic [25]. Therefore, detection of antibodies (HI-titer ≥10) after the pandemic is probably a good proxy for infection in the unvaccinated women in our cohort. However, a small proportion in our cohort may have had cross-reactive antibodies against A(H1N1)pdm09 from previous influenza infections. On the other hand, due to HI-titer waning [33], some of the infected women may have had undetectable levels of antibodies as the mean time between the pandemic and blood sampling was 6 months. However, this time interval was adjusted for in the analyses.

Few of the women in our study experienced adverse birth outcomes. We observed 41 cases of PTB among the 1258 women included in the analyses, corresponding to a rate of 3.3%. Among all the women who participated in the study, the rate of singleton PTB was 3.1%, which is lower than the average rate of 5.3% observed among live-born singletons in Norway in 2008 [34]. The lower rate may be explained by the NorFlu participants being more health conscious than the general population. This is supported by the high proportion of participants using folic acid during pregnancy (according to their record in the Medical Birth Registry) compared to all women in Norway giving birth in 2010 (71.5% vs. 27.0%) [35]. The participants in the NorFlu study may not be representative of the general population, but this does not necessarily result in biased effect estimates. A study on self-selection in MoBa found lower prevalence of several risk factors, pregnancy complications, and adverse birth outcomes among participants than among all women giving birth in Norway [36]. However, this study found no evidence of bias in exposure-outcome associations. Recruitment to NorFlu was based on the experience from MoBa and followed the same procedures, thus we expect this to be true for the NorFlu study also.

Even though the information on the outcomes was almost complete in this study, and only a handful of the participants were lost to follow-up, we did not have complete information on the exposures. More than 10% of the unvaccinated women did not return the influenza questionnaire and were therefore excluded. Among these women, the rate of singleton PTB was 6.0%, considerably higher than among the women included in the study. Furthermore, women without a blood sample who were excluded from the analyses with HI-titer as the main exposure, had a higher rate of singleton PTB than women included in these analyses (4.6% vs 2.4%). This indicates that the included participants tend to be healthier than the excluded participants. It is possible that influenza infection has a more harmful effect on birth outcomes for less healthy individuals with certain underlying conditions. In that case, the selection will probably have resulted in an underestimation of the effects of influenza during pregnancy.

In previous studies, mild A(H1N1)pdm09 influenza during pregnancy was not associated with mean birth weight [7, 16] or increased risk of PTB [14–16], SGA birth [7, 14, 16], or birth weight < 2500 g [7, 14–16]. This is in correspondence with our results. Risks of hospitalization and death due to influenza are highest in the third trimester [37], but whether the timing of influenza exposure during pregnancy is of importance in relation to birth outcomes is not clear. In a recent study from Canada, no increased risk of PTB was observed for women with medically attended pandemic influenza in their first or second trimester [18]. Influenza in the third trimester was associated with significantly increased risk of PTB, but only in the subgroup of women belonging to an influenza risk group. Since recruitment to our study started in February 2010, the majority of the participants were in their first trimester during the main wave of the pandemic. Thus, we could not study whether the impact of influenza differs with trimester of exposure.

Influenza and detection of A(H1N1)pdm09 antibodies were not significantly associated with risk of pre-eclampsia in our study. As far as we are aware, only two studies have previously investigated A(H1N1)pdm09 influenza during pregnancy and risk of pre-eclampsia [6, 7]. In both studies, women with A(H1N1)pdm09 influenza were compared to women with suspected influenza who tested negative for A(H1N1)pdm09. In accordance with our results, no significant differences in the proportion with pre-eclampsia were observed in these studies.

Only a few, mainly older, studies have used serology to identify women infected with influenza during pregnancy, thus also capturing milder and asymptomatic influenza cases [38–41]. When comparing infected and uninfected mothers, none of these studies found significant differences in mean birth weight [39–41] or proportion with low birth weight [38]. Detection of antibodies was not associated with median birth weight in our study. However, the 10th percentile of birth weight was more than 100 g lower for women with HI-titer ≥10 than women with undetectable levels of antibodies. In the subgroup with birth weight in the lowest decile, HI-titer was not associated with gestational age. Thus, the difference in birth weight does not seem to be a result of lower gestational age among those with HI-titer ≥10. Possibly, influenza infection may have a direct effect on intrauterine growth. This is supported by randomized clinical trials (RCTs) on maternal influenza vaccination [42, 43]. In RCTs, a higher risk of adverse birth outcomes among unvaccinated women can be attributed to the higher incidence of influenza infection. In line with our results, the RCTs found that women who received influenza vaccine had lower risk of low birth weight compared to women in the control group, although a significant difference in mean birth weight was also observed. In our study, a high proportion of women with HI-titer ≥10 and birth weight in the lowest decile belonged to an influenza risk group (19.2%). This may indicate that influenza infection has a stronger effect on birth weight for mothers with underlying conditions. Whether the offspring of less healthy pregnant women are more susceptible to the harmful effects of influenza infection should be further studied.

## Conclusions

Very few women in this population-based cohort of women who were pregnant during the 2009 pandemic had severe influenza. Furthermore, we found little evidence that mild influenza during pregnancy is associated with increased risk of pre-eclampsia, PTB or SGA birth. However, our findings may suggest that even mild or asymptomatic influenza infection during pregnancy may reduce the birth weight of the smallest children.

## Abbreviations

CI: Confidence interval
MBRN: The Medical Birth Registry of Norway
NorFlu: The Norwegian Influenza Pregnancy Cohort
OR: Odds ratio
PTB: Preterm birth
SGA: Small for gestational age
